# Prescriptive appropriateness of echocardiography for the diagnosis of infective endocarditis: an 11-year observational study

**DOI:** 10.1093/ehjci/jeaf306

**Published:** 2025-11-03

**Authors:** Antonella Cecchetto, Giulia Baroni, Angela Stievano, Stefano Nistri, Giovanni Borile, Donato Mele

**Affiliations:** Cardiology Unit, University of Padua-Azienda Ospedaliera, Via Giustiniani 2, 35128 Padua, Italy; Department of Cardio-Thoraco-Vascular Sciences and Public Health, University of Padua, Via Giustiniani 2, 35128 Padua, Italy; Department of Cardio-Thoraco-Vascular Sciences and Public Health, University of Padua, Via Giustiniani 2, 35128 Padua, Italy; Department of Cardio-Thoraco-Vascular Sciences and Public Health, University of Padua, Via Giustiniani 2, 35128 Padua, Italy; Department of Cardio-Thoraco-Vascular Sciences and Public Health, University of Padua, Via Giustiniani 2, 35128 Padua, Italy; Cardiology Service, CMSR Veneto Medica, Via Vicenza 204, 36077 Altavilla Vicentina, Italy; UOC Sistemi Informativi, University of Padua-Azienda Ospedaliera, Via Giustiniani 2, 35128 Padua, Italy; Cardiology Unit, University of Padua-Azienda Ospedaliera, Via Giustiniani 2, 35128 Padua, Italy; Department of Cardio-Thoraco-Vascular Sciences and Public Health, University of Padua, Via Giustiniani 2, 35128 Padua, Italy

**Keywords:** endocarditis, diagnosis, echocardiography, appropriateness

## Abstract

**Aims:**

Transthoracic (TTE) and transoesophageal echocardiography (TEE) are fundamental tools in diagnosing infective endocarditis (IE). Although IE remains rare, ultrasound (US) requests are increasing. No long-term data exist regarding the appropriateness of US prescriptions for IE following the 2017 Appropriate Use Criteria (AUC) for Multimodality Imaging in Valvular Heart Disease.

**Methods and results:**

US requests for suspected IE from September 2013 to June 2024 were reviewed. Patient records were retrieved electronically. Appropriateness was assessed using the 2017 AUC, the 2015 ESC Guidelines for IE management, and the 2013 Guidelines for TEE performance. Over 11 years, 2461 US requests, each referring to a unique hospitalized patient, were analysed. Most patients were males (60.5%), mean age 64 ± 17 years. Positive blood cultures were found in 41.8%, and IE was diagnosed in 10.6%, with a mortality rate of 7.6%. Overall, 1559 (63.4%) US requests were deemed inappropriate, with no significant change after guideline publication (*P* = 0.078). Specifically, 1402 (64.8%) TTE and 157 (52.7%) initial TEE requests were inappropriate. When TEE was used as a supplemental test, 138 (61.9%) were technically appropriate and 122 (54.7%) clinically appropriate. Cardiologists submitted more appropriate requests (65.8%) than non-cardiologists. Among appropriate requests, IE was confirmed in 15.3% of cases.

**Conclusion:**

Most US requests for suspected IE were inappropriate, particularly those made by non-cardiologists, highlighting the need for improved adherence to imaging guidelines, with potential benefits for patient care and resource management.

## Structured Graphical Abstract

**Graphical Abstract:**
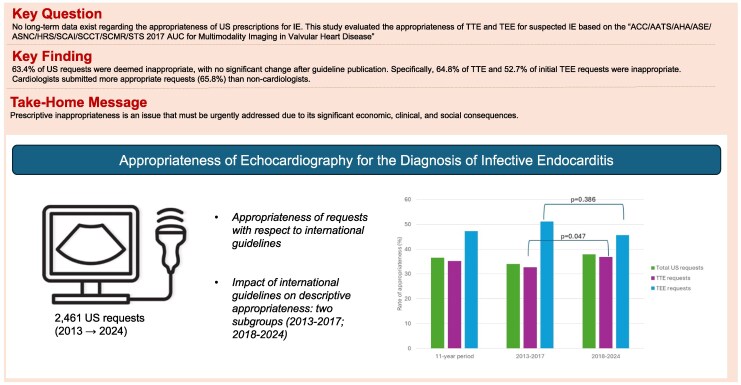

## Introduction

Infective endocarditis (IE) is rare but increasingly common (13.8 cases per 100 000/year),^1^ due to longer life expectancy, better diagnostics, more surgical procedures, and improved healthcare access.

The latest ESC guidelines for IE diagnosis combine clinical, microbiological, and imaging criteria.^2^ Transthoracic echocardiography (TTE) is usually the first test, but transoesophageal echocardiography (TEE) is recommended if TTE is inconclusive, negative with strong clinical suspicion, or positive but needing evaluation of complications. TEE may also be the initial choice in patients with prosthetic valves or intracardiac devices. The decision to use ultrasound (US) depends on pre-test probability, which is high when patients present with fever, positive blood cultures, and risk factors, especially if no other infection source is found.^3,4^

Despite the low incidence of IE, there is a high volume of daily in-hospital requests for TTE and TEE to diagnose this condition. Over-prescription of US can lead to a significant strain on human, economic, material, and managerial hospital resources. Consequently, it is crucial to systematically monitor the appropriateness of echocardiographic requests in suspected IE to avoid unnecessary and potentially harmful investigations.

Few studies have examined the use of echocardiography in relation to released appropriateness criteria for echocardiography. Focusing on US requested for suspected IE, 79% of adult inpatient requests were deemed appropriate and 13% inappropriate according to the 2007 Appropriate Use Criteria (AUC) in one study conducted by the general medicine service at a tertiary referral centre.^5^ Currently, there are no data in the Literature regarding the appropriateness of US prescriptions in the diagnosis of IE during a long period of observation and following the publication of the ‘*ACC/AATS/AHA/ASE/ASNC/HRS/SCAI/SCCT/SCMR/STS 2017 AUC for Multimodality Imaging in Valvular Heart Disease’*.^6^

Therefore, the primary aim of this study was to evaluate the appropriateness of echocardiographic prescriptions—both TTE and TEE—for suspected IE based on the ‘*ACC/AATS/AHA/ASE/ASNC/HRS/SCAI/SCCT/SCMR/STS 2017 AUC for Multimodality Imaging in Valvular Heart Disease’*.^6^ Secondary objectives included analysing the impact of international guidelines on the appropriateness of these requests and exploring the relationship between prescriptive appropriateness and the category of prescriber. When appropriate, educational interventions aimed at physicians should be implemented to increase the rate of correct TTE and TEE utilization.^7^

## Methods

This is an observational, retrospective, single-centre study, with data collected between September 2013 and June 2024.

### Ethical considerations

The study was approved by the Ethics Committee of the University Hospital of Padua (Protocol No. 264n/AO/22) and was conducted in full compliance with human dignity and fundamental rights, in accordance with the principles of the Declaration of Helsinki.^8^

### Study population and data sources

**Figure 1:**
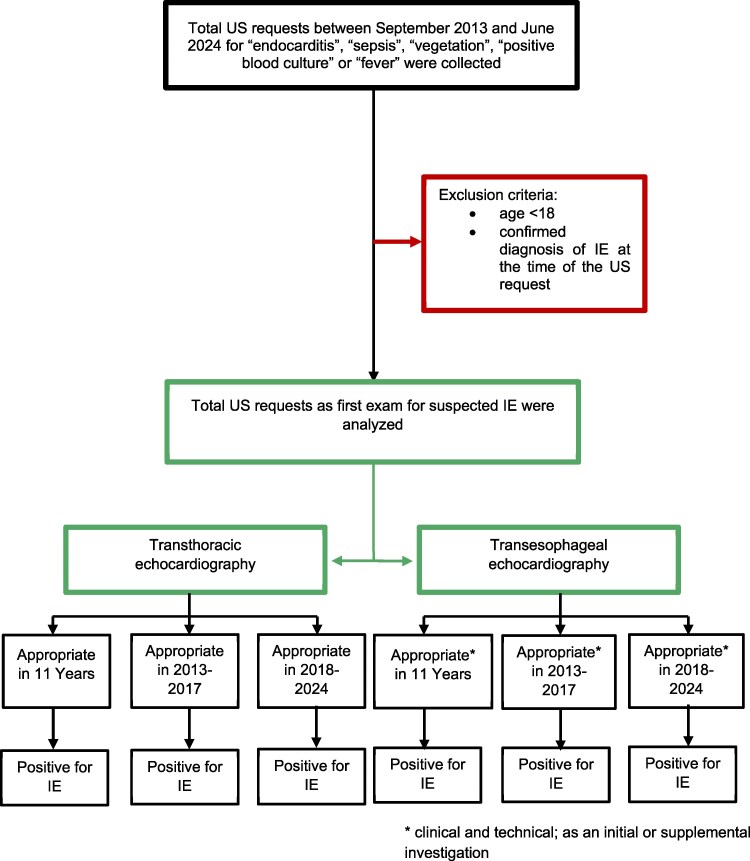
Flow chart of the study.

US requests for suspected IE were retrospectively collected from September 2013 to June 2024 at the University Hospital of Padua. To identify relevant cases, searches were conducted within the echocardiographic database using diagnostic keywords such as ‘endocarditis’, ‘fever’, ‘vegetation’, ‘positive blood culture’, and ‘sepsis’. Each US request was subsequently reviewed to confirm its association with suspected IE. Only initial requests for TTE or TEE were included in the primary analysis. For each identified patient, data were extracted from electronic medical records, including demographic characteristics, clinical manifestations, microbiological findings, additional imaging studies [e.g. computed tomography (CT), positron emission tomography (PET)], in-hospital outcomes, and discharge diagnoses based on the International Classification of Diseases, Ninth Revision (ICD-9). The professional category of the requesting physician was also recorded for each US request. Inclusion criteria were age >18 years and hospitalization at the time of the request. Exclusion criteria included a confirmed diagnosis of IE at the time of the US request and requests made for conditions not suggestive of IE. Subsequently, requests for TEE performed as a supplementary examination following an initial TTE were also collected and analysed. *Figure 1* illustrates the flowchart of the study.

### Assessment of prescriptive appropriateness

The prescriptive appropriateness of each identified US request was assessed in accordance with international guidelines. Specifically, clinical appropriateness was evaluated for TTE requests, while both clinical and technical appropriateness were assessed for TEE requests, whether performed as an initial or supplemental investigation.

To determine clinical appropriateness of TTE and TEE requests for suspected IE (both initial and supplemental), the 2017 ACC/AATS/AHA/ASE/ASNC/HRS/SCAI/SCCT/SCMR/STS AUC for Multimodality Imaging in Valvular Heart Disease^6^ was used. According to these criteria, a request was considered clinically appropriate if the patient had a suspected IE and positive blood cultures or a new murmur. In contrast, it was deemed inappropriate in cases of transient fever without bacteraemia or a new murmur, transient bacteraemia with a pathogen not typically associated with IE, and/or a documented non-endovascular source of infection.

Technical appropriateness of initial TEE requests was assessed based on the Guidelines for Performing a Comprehensive Transoesophageal Echocardiographic Examination by the American Society of Echocardiography and the Society of Cardiovascular Anaesthesiologists.^4^ A request was considered technically appropriate if: the patient was critically ill (where TTE was unlikely to yield sufficient diagnostic information); TTE was non-diagnostic; or TTE was deferred due to a high likelihood of being non-informative. Scenarios where TTE is likely to be non-diagnostic include detailed evaluation of distant structures (e.g. the aorta, left atrial appendage); assessment of prosthetic valves; suspected paravalvular abscesses (in both native and prosthetic valves); mechanically ventilated patients; patients with chest wall trauma; obesity or other body habitus limitations; and patients unable to assume the left lateral decubitus position. Requests not meeting these indications were classified as technically inappropriate.

For supplemental TEE requests, technical appropriateness was evaluated using the 2015 ESC Guidelines for the Management of IE.^9^ According to these recommendations, TEE is indicated in all patients with suspected IE and either a negative or non-diagnostic TTE, or when a prosthetic valve or intracardiac device is present (Class I, Level B). Additionally, TEE is recommended in patients with positive TTE findings, except in cases of isolated right-sided native valve IE with high-quality, unequivocal TTE imaging (Class IIa, Level C). Requests falling outside these indications were considered technically inappropriate.

### Impact of the international guidelines in the prescriptive appropriateness of TTE and TEE

To analyze the impact of the ‘ACC/AATS/AHA/ASE/ASNC/HRS/SCAI/SCCT/SCMR/STS 2017 AUC for Multimodality Imaging in Valvular Heart Disease’^6^ on the prescriptive appropriateness of TTE and TEE, before and after its publication, the study population was divided into two subgroups. The first subgroup consisted of US requests issued from 2013 to 2017, and the second subgroup consisted of US requests issued from 2018 to 2024.

### Statistical analysis

Continuous data were presented as mean ± standard deviation (SD), while categorical data were presented as counts and percentages. Comparisons of categorical variables were performed using the chi-square test, and comparisons of non-categorical variables were performed using the *t*-test. A *P*-value of <0.05 was considered statistically significant.

## Results

Between September 2013 and June 2024, a total of 2742 US requests for initial examinations were retrieved from medical records. After excluding duplicate requests, requests for patients with a confirmed diagnosis of IE, and those made for conditions unrelated to IE, a final cohort of 2461 US requests concerning the same number of hospitalized patients was included in the analysis.

The number of US requests during the 2013–2017 period was significantly lower compared to subsequent years (926 US requests during the 2013–2017 period vs. 1535 US requests during the 2018–2024 period, *P* < 0.00001), a trend observed for both TTE and TEE requests (*P* = 0.010). Over the 11-year study period, a total of 521 TEE requests were recorded, of which 298 (57.2%) were performed as initial investigations and 223 (42.8%) as supplemental examinations following an initial TTE.

### Population characteristics

The US requests analysed as initial examinations were predominantly for middle-aged male patients (60.5% male; mean age 64 ± 17 years). In the earlier subgroup, there was a significantly higher proportion of patients transferred from other hospitals (76%, *P* < 0.00001). The most frequent presenting symptom was fever (43.3%), and 46.8% of patients had an identified infection site other than the endocardium at the time of the request.

Regarding risk factors for IE, immunosuppression and the presence of central venous access were significantly more common in the later subgroup (*P* = 0.049 and *P* = 0.022, respectively). From a microbiological standpoint, 41.8% of patients had positive blood cultures, with 71.4% of these, positive for pathogens typically associated with IE. A significant increase in typical pathogen detection was observed in the later subgroup (*P* = 0.003). The most frequently isolated pathogen was *Staphylococcus* species (65.1%), with *Staphylococcus aureus* identified in 31.7% of cases.

IE was diagnosed in 260 patients (10.6%). Of these patients, 122 (46.9%) underwent cardiac surgery, and 414 (51%) required admission to the intensive care unit. The mean duration of hospitalization was 28 ± 26 days. In-hospital mortality was 7.6%.

Demographic data, clinical and microbiological characteristics, and patient outcomes are summarized in *Table 1*.

**Table 1:** Study population characteristics in an 11-year period, and before and after the publication of the ‘2017 international guidelines on appropriateness for cardiac imaging in valvular heart disease’

	11-year period (2013–2024)	Pre-2017 AUC (2013–2017)	Post-2017 AUC (2018–2024)	*P*-value (pre- vs. post-2017 AUC)
**Patients (*n*)**	2461	926	1535	
**Demographics**				
Age (years, mean ± DS)	64 ± 17	63 ± 18	64 ± 17	0.645
Male *n* (%)	1490 (60.5%)	553 (59.7%)	937 (61.0%)	0.515
Transferred from other hospital *n* (%)	1095 (44.5%)	704 (76%)	391 (25.7%)	**<0**.**00001**
**Clinical findings *n* (%)**				
Fever	1069 (43.4%)	416 (44.9%)	653 (42.5%)	0.248
Cardiac murmur	113 (4.6%)	41 (4.4%)	72 (4.7%)	0.763
Known infection site	1152 (46.8%)	444 (47.9%)	708 (46.1%)	0.379
Previous IE	87 (3.5%)	36 (3.9%)	51 (3.3%)	0.462
Congenital heart disease	14 (0.6%)	5 (0.5%)	9 (0.6%)	0.882
Intravenous drug use	59 (2.4%)	23 (2.5%)	36 (2.4%)	0.828
Central venous access	123 (5.0%)	36 (3.9%)	87 (5.7%)	**0**.**049**
Intracardiac device	47 (1.9%)	13 (1.4%)	34 (2.2%)	0.154
Prosthetic valve	191 (7.8%)	75 (8.1%)	116 (7.6%)	0.626
Immunosuppression	269 (10.9%)	84 (9.1%)	185 (12.1%)	**0**.**022**
Haemodialysis	77 (3.1%)	28 (3.0%)	49 (3.2%)	0.816
Immunological events	16 (0.6%)	5 (0.5%)	11 (0.7%)	0.597
Vascular events	62 (2.5%)	22 (2.4%)	40 (2.6%)	0.724
Recent surgery	135 (5.5%)	51 (5.5%)	84 (5.5%)	0.970
**Microbiological findings *n* (%)**				
Positive blood culture	1028 (41.8%)	378 (40.8%)	650 (42.4%)	0.457
• Of them, positive for typical pathogen	734 (71.4%)	248 (65.9%)	485 (74.6%)	**0.003**
Most frequent pathogen isolated	*Staphylococcus* 478 (65.1%), *S. aureus* 233 (31.7%)	*Staphylococcus* 148 (59.7%), *S. aureus* 71 (28.6%)	*Staphylococcus* 330 (68%), *S. aureus* 162 (33.4%)	**0.025**, 0.189
**Outcome *n* (%)**				
EI diagnosis	260 (10.6%)	83 (8.9%)	177 (11.5%)	**0.047**
• Of them, patients undergoing cardiac surgery	122 (46.9%)	38 (45.8%)	84 (47.5%)	0.801
Transfers during hospitalization	813 (33%)	273 (29.5%)	540 (35.2%)	**0**.**004**
• Of them, in surgery ward	382 (47%)	130 (47.6%)	252 (46.7%)	0.797
• Of them, in intensive care unit	414 (51%)	139 (50.9%)	275 (50.9%)	0.998
**Discharge**				
Days of hospitalization (mean ± DS)	28 ± 26	27 ± 26	29 ± 26	0.213
Home discharge *n* (%)	1923 (78.1%)	736 (79.5%)	1187 (77.3%)	0.211
Transferred to other hospital *n* (%)	351 (14.3%)	119 (12.9%)	232 (15.1%)	0.199
In-hospital death *n* (%)	187 (7.6%)	71 (7.7%)	116 (7.6%)	0.920

*n*, number.

### Prescription appropriateness

**Figure 2:**
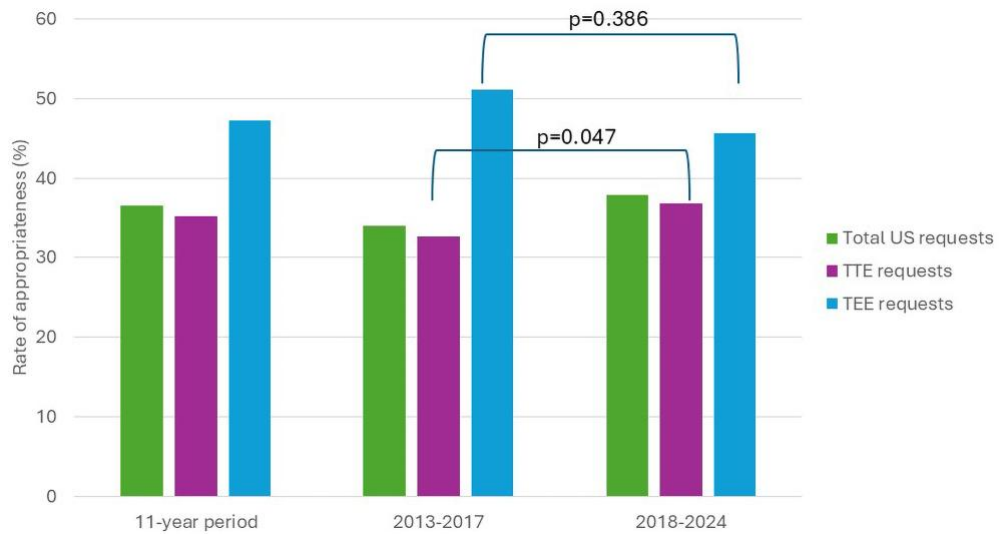
Rate of appropriateness of echocardiography requests in an 11-year period, and before and after the publication of the ‘2017 international guidelines on appropriateness for cardiac imaging in valvular heart disease’.

**Figure 3:**
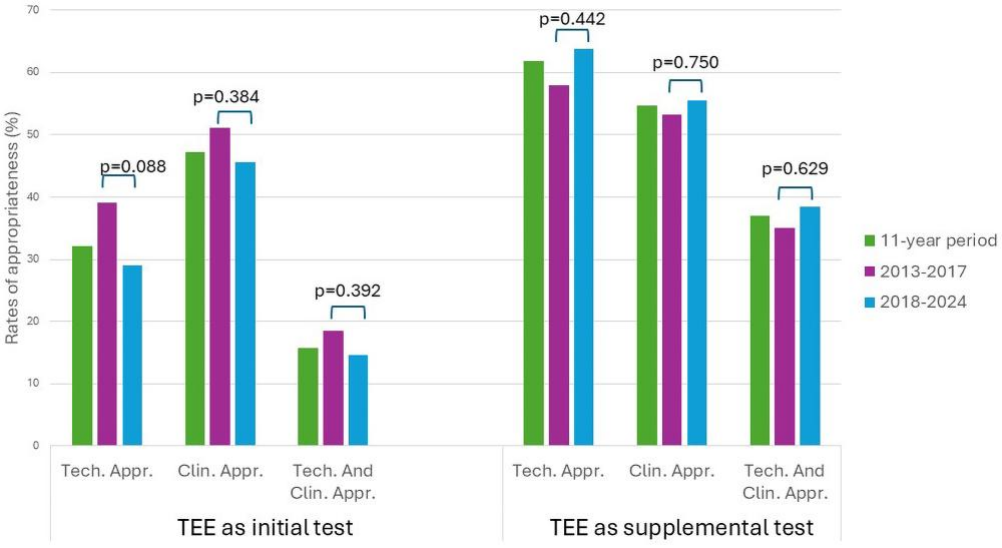
Rate of clinical and technical appropriateness of TEE exams performed as initial and supplemental test in an 11-year period, and before and after the publication of the ‘2017 international guidelines on appropriateness for cardiac imaging in valvular heart disease’. Clin.Appr = clinical appropriateness; Tech.Appr.= technical appropriateness.

Over the 11-year study period, 1559 of the 2461 US requests for initial examinations (63.4%) were classified as inappropriate. Among TTE requests, 35.2% met the criteria for clinical appropriateness. For TEE performed as an initial investigation, 47.3% of requests were deemed clinically appropriate, 32.2% met technical appropriateness criteria, and only 15.8% were considered both clinically and technically appropriate (*Figures 2* and *3*; *Tables 2* and *3*).

**Table 2:** Appropriateness of echocardiography requests and rate of diagnosis of IE in appropriate cases in an 11-year period, and before and after the publication of the ‘2017 international guidelines on appropriateness for cardiac imaging in valvular heart disease’

	11-year period (2013–2024) (*n*)	Pre-2017 AUC (2013–2017) (*n*)	Post-2017 AUC (2018–2024) (*n*)	*P*-value (pre- vs. post-2017 AUC)
**Total ultrasound requests as first exam (*n*)**	2461	926	1535	**<0.00001**
Of them, appropriate *n* (%)	902 (36.6%)	319 (34%)	583 (37.9%)	0.078
○ Of them, positive for IE *n* (%)	138 (15.3%)	40 (12%)	98 (16.8%)	0.088
**TTE requests (*n*)**	2163	834	1329	**0.010**
Of them, appropriate *n* (%)	761 (35.2%)	272 (32.6%)	489 (36.8%)	**0.047**
○ Of them, positive for IE *n* (%)	99 (13%)	32 (11.8%)	67 (13.7%)	0.447
**TEE requests (*n*)**	298	92	206	**0.010**
Of them, appropriate *n* (%)	141 (47.3%)	47 (51.1%)	94 (45.6%)	0.386
○ Of them, positive for IE *n* (%)	39 (27.7%)	8 (17%)	31 (32.9%)	**0.046**

*n*, number.

**Table 3:** Technical and clinical appropriateness of transoesophageal exams performed both as initial and supplemental investigation in an 11-year period, and before and after the publication of the ‘2017 international guidelines on appropriateness for cardiac imaging in valvular heart disease’

	11-year period (2013–2024)	Pre-2017 AUC (2013–2017)	Post-2017 AUC (2018–2024)	*P*-value (pre- vs. post-2017 AUC)
Total TEE (*n*)	521	169	352	
TEE as initial test *n* (%)	298 (57.2%)	92 (54%)	206 (58.5%)	0.378
Technically appropriate	96 (32.2%)	36 (39.1%)	60 (29.1%)	0.088
Clinically appropriate	141 (47.3%)	47 (51.1%)	94 (45.6%)	0.384
Technically and clinically appropriate	47 (15.8%)	17 (18.5%)	30 (14.6%)	0.392
TEE as supplemental test *n* (%)	223 (42.8%)	77 (45.6%)	146 (41.5%)	0.378
Technically appropriate	138 (61.9%)	45 (58%)	93 (63.7%)	0.442
Clinically appropriate	122 (54.7%)	41 (53.3%)	81 (55.5%)	0.750
Technically and clinically appropriate	83 (37%)	27 (35.1%)	56 (38.4%)	0.629

*n*, number.

When TEE was performed as a supplemental examination following TTE, 54.7% of requests were clinically appropriate, 61.9% were technically appropriate, and 37.0% met both clinical and technical appropriateness standards (*Figure 3*; *Table 3*).

Among patient subgroups with a high baseline risk for IE, US prescription appropriateness was higher in those with valve prostheses (48%) and in patients with cardiac implantable electronic devices (57%), whereas it was lower in individuals with a history of drug abuse (25%).

### Impact of guidelines on prescriptive appropriateness

No statistically significant difference was observed in the overall rate of appropriate US prescriptions before and after the publication of the 2017 international guidelines (*P* = 0.078) (*Figure 2*; *Table 2*).

However, when analysing TTE requests specifically, a statistically significant increase in clinical appropriateness was observed following the release of the guidelines, with appropriateness rising from 32.6% in the pre-guideline period to 36.8% in the post-guideline period (*P* = 0.047). (*Figure 2*; *Table 2*).

In contrast, no significant differences were found between the two periods for TEE requests performed as initial examinations, whether assessed from a clinical (*P* = 0.386), technical (*P* = 0.088), or combined clinical and technical (*P* = 0.392) standpoint, despite a trend towards reduced appropriateness (*Figure 3*; *Table 3*).

Regarding TEE performed as a supplemental evaluation, an increase in the proportion of clinically, technically, and combined appropriate requests was observed in the 2018–2024 period; however, none of these differences reached statistical significance (*P* = 0.750, *P* = 0.442, and *P* = 0.629, respectively) (*Figure 3*; *Table 3*).

No significant changes in appropriate US prescription rates were observed in high-risk patient subgroups following the 2017 international guidelines (valve prostheses, *P* = 0.74; cardiac implantable electronic devices, *P* = 0.76; drug users, *P* = 0.60).

### Rate of diagnosis of IE in appropriate and inappropriate requests

Among the appropriate US requests over the 11-year study period, 138 cases (15.3%) resulted in a confirmed diagnosis of IE. The diagnostic yield was higher for TEE, with 27.7% of appropriate requests leading to an IE diagnosis, compared with 13.0% of TTE (*Table 2*).

When analysing the two study subperiods, a modest but statistically significant increase in the diagnostic yield of appropriate TEE requests was observed in the 2018–2024 period (*P* = 0.046). In contrast, no significant difference was found for TTE across the two timeframes (*P* = 0.477).

Among all inappropriate US, the rate of IE diagnosis was 7.8%.

### Relationship between appropriateness and prescribers’ categories

**Figure 4:**
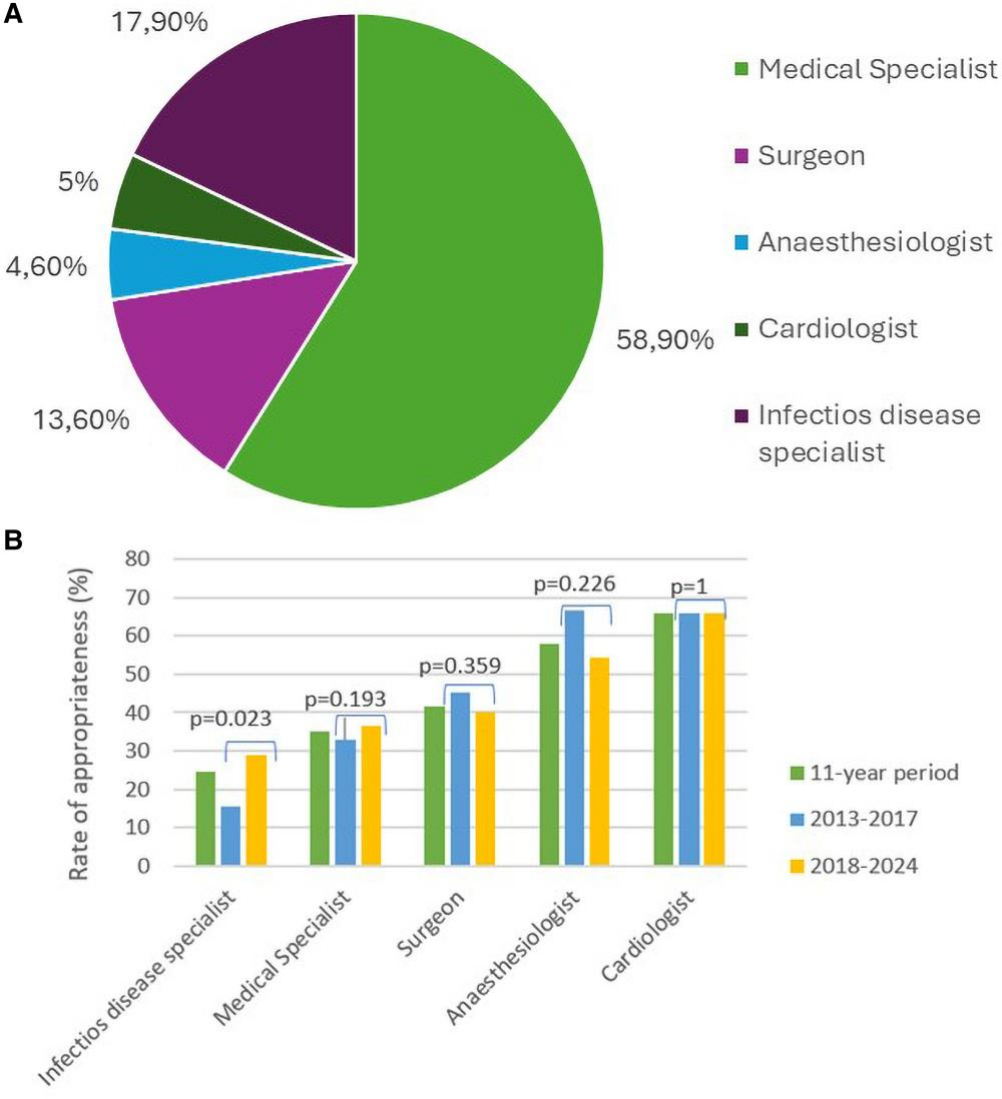
A percentages of US requests depending on the category of ordering physician in the 2013–2024 period. B Rate of appropriateness of US requests divided by ordering physicians in an 11-year period, and before and after the publication of the ‘2017 international guidelines on appropriateness for cardiac imaging in valvular heart disease’.

Over the 11-year study period, the highest proportion of US requests for suspected IE came from internal medicine specialists (58.9%), followed by infectious disease specialists (17.9%), surgeons (13.6%), cardiologists (5%), and anaesthesiologists (4.6%) (*Figure 4*). When comparing the two subperiods, a statistically significant increase in requests was observed from surgeons (*P* = 0.028) and infectious disease specialists (*P* = 0.011), while a significant decrease was noted for internal medicine specialists (*P* < 0.00001).

The most appropriate prescriptions were made by cardiologists (65.8%), followed by anaesthesiologists (57.9%). Among the prescriber categories, only prescriptions made by infectious disease specialists showed a statistically significant increase in appropriateness during the 2018–2024 period (*P* = 0.002). No significant differences were observed in the appropriateness of prescriptions from the other specialities between the two subperiods (*Table 4*).

**Table 4:** Total number and rate of appropriateness of US requests divided by ordering physicians in an 11-year period, and before and after the publication of the ‘2017 international guidelines on appropriateness for cardiac imaging in valvular heart disease’

	11-year period (2013–2024) *n* (%)	Pre-2017 AUC (2013–2017) *n* (%)	Post-2017 AUC (2018–2024) *n* (%)	*P*-value (pre- vs. post-2017 AUC)
Medical specialist	1449 (58.9%)	602 (65%)	847 (55%)	**<0.00001**
Of them, appropriate	507 (35%)	199 (33%)	308 (36.4%)	0.193
Surgeon	335 (13.6%)	108 (11.7%)	227 (14.8%)	**0.028**
Of them, appropriate	140 (41.8%)	49 (45.4%)	91 (40%)	0.359
Anaesthesiologist	114 (4.6%)	33 (3.6%)	81 (5.3%)	0.051
Of them, appropriate	66 (57.9%)	22 (66.7%)	44 (54.3%)	0.226
Cardiologist	123 (5%)	41 (4.4%)	82 (5.3%)	0.313
Of them, appropriate	81 (65.8%)	27 (65.9%)	54 (65.8%)	1
Specialist in infectious disease	440 (17.9%)	142 (15.3%)	298 (19.4%)	**0.011**
Of them, appropriate	108 (24.5%)	22 (15.5%)	86 (28.9%)	**0.0023**

*n*, number.

## Discussion

This study evaluated for the first time the appropriateness of US prescriptions in the diagnosis of IE during a long period of observation and following the publication of the ‘*ACC/AATS/AHA/ASE/ASNC/HRS/SCAI/SCCT/SCMR/STS 2017 AUC for Multimodality Imaging in Valvular Heart Disease’.* This study demonstrated several important findings related to the prescription of TTE and TEE in the diagnosis of IE. Over the years, there has been a progressive increase in the number of TTE and TEE requests for IE. However, the overall clinical appropriateness of these requests was relatively low, with only about one-third considered appropriate. The appropriateness rate was higher for TEE compared with TTE. Despite the publication of international guidelines aimed at optimizing echocardiography use, there was no significant change in prescribing appropriateness, except for TTE, where a slight improvement was noted. Additionally, cardiologists demonstrated greater prescribing appropriateness compared with other specialists.

The study population showed characteristics consistent with existing literature on IE. Most patients were middle-aged men, though IE is more common in older groups.^1^ Risk factors included valve prosthesis, intracardiac devices, haemodialysis, intravenous drug use, and immunosuppression,^10,11^ with the latter more frequent in recent years due to the centre’s tertiary care profile. The observed mortality rate (7.6%) was lower than in previous studies,^10,11^ possibly due to a lower percentage of confirmed IE cases and improved access to specialized care and cardiac surgery.

The study found a progressive rise in TTE and TEE requests for suspected IE. While full appropriateness is unrealistic, indiscriminate ‘blanket screening’ is unjustified, leading to false positives, unnecessary treatments, and further invasive tests. Inappropriate requests also increase healthcare costs, prolong hospitalizations, strain outpatient services, delay urgent exams, and contribute to clinician workload, errors, and burnout.

The first document addressing the issue of appropriateness in echocardiography was published in 2007^12^ and updated in 2011.^13^ Following their publication, several studies examined the application of AUC for TTE in both inpatient and outpatient settings.

Ballo *et al*., referencing the 2011 AUC, found that 80.3% of TTE requests for hospitalized patients during a 6-week period were appropriate. The inappropriate requests were more frequently ordered by non-cardiologists and younger physicians.^14^ Morrone *et al*. focused on outpatient settings over a 6-month period, reporting an inappropriateness rate of 11% for TTE requests, again mostly ordered by non-cardiologists.^15^ Conversely, a study conducted at Parma University Hospital over 1 year reported a much higher inappropriateness rate (41.5%) for outpatient TTE requests.^16^ This was attributed to the lack of systematic monitoring within the Italian national health system. Although most TTEs were prescribed by general practitioners, the rates of inappropriate requests were similar between cardiologists and non-cardiologists. Notably, Gaibazzi *et al*. were unable to differentiate between TTEs requested by hospital-based cardiologists and those working in community settings.^16^ Fonseca *et al*., over a 1-month period, found that 78.7% of TTE requests (both inpatient and outpatient) were appropriate. In contrast to the Italian studies, cardiologists—particularly residents—had a higher rate of inappropriate requests than specialists.^17^ In the United States, Martin *et al*. reported that 86% of inpatient TTE requests over a 35-day period were appropriate, according to the 2007 AUC.^5^ Kirkpatrick *et al*., also referencing the 2007 AUC, found that during a 2-month period, 56% of outpatient TTEs were appropriate. The relatively low rate of inappropriate requests may have been due to the fact that cardiologists ordered the majority (53%) of the exams.^18^ In a Saudi Arabian study by Alotaibi *et al*., 77.9% of TTE requests made over 1 month were appropriate. Most of the requests came from the Department of Medicine, with anaesthesiologists (for outpatient exams) and surgeons (for inpatient exams) accounting for a significantly higher proportion of inappropriate requests.^19^

In summary, as evidenced by the cited studies, there is considerable variability in appropriateness rates for TTE requests, ranging from 56% to 86%, and inappropriateness rates from 8% to 41.5%. These discrepancies can be attributed to differences in study settings (academic vs. non-academic hospitals, tertiary vs. non-tertiary centres), patient populations (inpatient vs. outpatient), the relatively short timeframes of the studies, and challenges in applying AUC due to variability in access to patient information.

Focusing on TTEs requested for suspected IE, 79% of adult inpatient requests were deemed appropriate and 15% inappropriate according to the 2007 AUC in one study. The appropriateness rate improved when cardiologist consultation was involved.^5^ In our study, only 36.6% of US requests for suspected IE were appropriate—significantly lower than the rate reported by Martin *et al*.^5^ This may be due to the setting of our study—a tertiary academic centre—where we could not distinguish between requests made by residents and those by specialists. Additionally, the lack of systematic monitoring for testing appropriateness, neither mandated nor planned by the Italian national health system, further exacerbates the issue. Medico-legal pressure may contribute to inappropriate echocardiography requests, as defensive medicine leads clinicians to order imaging even with low pre-test probability. Although not assessed in this study, future research should examine the influence of legal liability on prescribing behaviour. Moreover, higher rates of inappropriate exams were ordered by non-cardiologists, who are typically less familiar with dedicated cardiovascular guidelines.

In 2011, Rahimi *et al*.^20^ conducted a similar study, examining TTE request trends before and after the release of the 2007 American College of Cardiology Foundation AUC.^11^ They found no significant change in the proportion of inappropriate referrals in the academic medical centre setting despite the publication of the criteria.^12^ In our study, we assessed for the first time the impact of international guidelines aimed at optimizing echocardiographic use in diagnosing IE. No significant change in appropriateness was observed after their publication, with the exception of a slight improvement noted in TTE requests.

Over the 11-year observation period, most echocardiography requests came from internal medicine physicians. While the number of requests increased for nearly all specialties, internists showed a decrease in requests between 2018 and 2024—possibly due to the COVID-19 pandemic, which primarily affected medical wards and reduced non-urgent testing to limit patient movement within hospitals. During the same period, we observed a significant increase in both the number and appropriateness of requests made by infectious disease specialists, although their overall appropriateness rate remained the lowest among all specialties.

Overall, the highest rates of appropriate TTE requests were observed among cardiology specialists, consistent with previous literature.^14,15^ This trend may be partly explained by the 2015 ESC Guidelines for the management of endocarditis,^9^ which include more accessible and specific appropriateness criteria than the more technical 2017 international guidelines on echocardiographic testing.

The rate of IE diagnosis in appropriate exams was about twice as high as in inappropriate ones, demonstrating the usefulness of guidelines in identifying patients who merit further diagnostic evaluation. However, the presence of disease even in patients without an indication for additional testing suggests that the guidelines fail to fully address grey areas.

### Potential solutions to improve the rate of appropriateness

The study showed that TEE represented nearly one-sixth of all echocardiographic requests, but only about half were appropriate. Given that TEE, though safe, is invasive and carries risks, its use should be carefully optimized.

Sivak *et al*. showed that applying strict criteria to define a negative TTE, rather than only excluding vegetations, can enhance its diagnostic reliability. In patients with low clinical suspicion for IE, this approach may reduce the need for unnecessary follow-up TEE.^21^

Additionally, physician oversight and stricter review of echocardiography indications have proven beneficial. For example, during the COVID-19 pandemic, the University of Chicago implemented a review strategy aimed at minimizing virus exposure. This resulted not only in a reduction in TTE volume but also in a significant increase in the appropriateness of performed exams.^22^

To curb the waste of financial and clinical resources, several countermeasures have been proposed. Bathia *et al*. showed that even simple educational interventions—such as lectures on the 2011 AUC, pocket reference cards with appropriate indication guidelines, and bi-weekly feedback on ordering practices—significantly reduced the rate of inappropriate TTEs, especially in the context of IE.^7^

Based on these findings, a comprehensive corrective strategy should include several components:

1. Periodic monitoring and AUC adoption

Regular audits of echocardiographic request appropriateness and systematic implementation of AUC can be effective. A systematic review by Kerley *et al*. confirmed that AUC are applicable beyond the US context. Their application across five international hospitals revealed that nearly one-fifth of TTEs could have been avoided.^23^ Similarly, Williams *et al*. found that cardiologist verification of echocardiography requests for suspected IE significantly reduced unnecessary testing.^24^

2. Development of shared in-hospital protocols

Establishing standardized protocols, agreed upon by all departments, can support decision-making in cases of clinical uncertainty.

3. Improving guideline awareness among non-cardiologists

The minimal change in appropriateness following guideline publication suggests that passive guideline dissemination is insufficient. Targeted educational interventions and decision-support tools are likely necessary to improve adherence, particularly among non-cardiologists who may rely on speciality-specific clinical frameworks. Notably, the 2023 ESC Guidelines for the management of endocarditis^2^ introduced specific risk scores for patients with positive blood cultures for *S. aureus*, *Enterococcus faecalis*, and *Streptococcus* species. These scores incorporate both microbiological parameters and cardiac-related risk factors, helping identify patients most likely to benefit from in-depth echocardiographic evaluation.

4. Review of appropriateness criteria

Finally, the high rate of inappropriate prescribing may reflect not only gaps in knowledge but also the complexity and rigidity of current appropriateness criteria. Guidelines should ideally balance evidence-based rigour with practicality, ensuring they can be consistently applied in everyday clinical practice. In addition to widespread dissemination, prospective evaluation of guideline applicability should be systematically conducted to identify barriers to implementation and refine recommendations for real-world use.

### Limitations of the study

This study has several limitations. First, it is retrospective, single-centre, and based in a tertiary Italian hospital, which may limit generalizability. Use of internationally accepted guidelines supports broader relevance, but reliance on keyword-based data and binary appropriateness classifications risks overlooking clinical nuances and causing selection bias. In high-complexity settings, many patients may fall into a grey zone that does not align clearly with binary appropriate/inappropriate classifications. Differences between the 2017 AUC criteria and the newer ESC/EACVI guidelines^2,3^ further highlight variability in practice. Finally, the study could not evaluate the impact of inappropriate echocardiography on clinical outcomes, underscoring the need for prospective, detailed data collection.

## Conclusions

Despite the critical role of TTE and TEE in diagnosing IE, most US requests in this study were inappropriate, particularly among non-cardiologists. This high rate likely reflects both knowledge gaps and the complexity of current appropriateness criteria. These findings highlight the need for targeted interventions—such as clinician education, institutional audit and feedback mechanisms, or integration of decision support tools—to enhance the appropriate use of echocardiography in suspected IE. Improved adherence to guidelines and prospective evaluation of their applicability could support better patient outcomes and more efficient use of healthcare resources.

## Data Availability

The datasets generated and/or analysed during the current study are available from the corresponding author on reasonable request.
